# Enhancement of thermo-mechanical stability for nanocomposites containing plasma treated carbon nanotubes with an experimental study and molecular dynamics simulations

**DOI:** 10.1038/s41598-019-56976-w

**Published:** 2020-01-15

**Authors:** Hana Jung, Hoi Kil Choi, Yuna Oh, Hyunkee Hong, Jaesang Yu

**Affiliations:** Composite Materials Application Research Center, Institute of Advanced Composite Materials, Korea Institute of Science and Technology (KIST), Chudong-ro 92, Bongdong-eup, Wanju-gun, Jeollabukdo 55324 Korea

**Keywords:** Engineering, Materials science

## Abstract

This study investigated differences in the thermo-mechanical properties of thermosetting polymer EPON 826 nanocomposites reinforced by modified nanofillers. Carbon nanotubes (CNTs) were modified by environmentally friendly plasma treatments. Composites containing various nitrogen doped CNTs were investigated by morphological and structural analysis, which confirmed that they provided better dispersion and stronger interfacial interaction with the epoxy matrix. In addition, the dynamic mechanical behavior and thermal conductivity were analyzed to understand the energy transfer mechanism in the nanocomposites. The thermal and mechanical properties of the Inductively coupled plasma treated CNTs (ICP-CNT) reinforced nanocomposites containing a high concentration of quaternary and pyridinic types were higher than that of mechanical shear force plasma treated CNTs (MSF-CNT). A molecular dynamics (MD) simulation was performed to support the experimental results and confirmed that controlling the type of nitrogen doping groups was important for improving the thermo-mechanical characteristics of CNT/epoxy nanocomposites.

## Introduction

Polymer composites have attracted significant attention in light emitting diode package, which needs to provide high-thermal conductivity and appropriate mechanical properties. Many studies have focused on the production of electrical and thermal conductive polymer composites using multiple polymers as a matrix and fillers such as modified carbon nanotubes (CNTs)^1–3^. CNT reinforced epoxy composites are extensively used in a large number of applications where a material with that combination of properties is needed because they have relatively high strength, modulus, and heat conduction^4^. The strength and thermal conductivity of the CNT composites are 25 and 40% higher than those of the composites containing 2D carbon materials^2^. However, in many cases, the full potential applications of CNT composites are presently limited because the CNTs become easily entangled and agglomerated because of their size and large aspect ratio^5–7^, which affect performance. One solution for this problem includes exploring composite methods, which can result in a uniform dispersion of the CNTs in the polymer matrix^8^. According to theories on the strength, modulus, and thermal conductivity of polymer composites, post-processing CNT distribution within the polymer matrix is one of the key factors that determine physical properties of the composites. Moreover, significant efforts have been attempted towards enhancing the thermal and mechanical properties on the composites through modified surface of CNTs, including the use of dopants^9–17^. Among the various potential dopants, nitrogen is presently considered as an excellent doping component for carbon-based materials. Nitrogen doping has been successfully utilized to alter the electrical and structural properties of CNTs^18^. Molecular dynamics (MD) simulation is a powerful tool for exploring the properties of materials at an atomic level and has been used to predict the mechanical and thermal properties of nanocomposites. For example, Choi *et al*.^19^ calculated Young’s modulus of epoxy structures with decreasing degrees of cross-linking and with increasing temperature. Kumar *et al*.^20^ characterized the corrosion resistance, reactivity, and stability of nitrogen doped graphene oxide coating on substrates. Sun *et al*.^21^ reported that oxygen-containing functional groups influenced the thermal conductivity of the reduced graphene oxide. Although theoretical studies have been performed to study the characteristics of modified nanofillers, the research on mechanism analysis for composites incorporating modified nanofillers is still lacked. In this study, doping effects on the structure and morphology of nanocomposites containing CNTs are in detail investigated to support a better understanding of the roles and design of environmentally friendly modifications.

## Results

### Morphology and dispersion stability of the nitrogen doped MWCNTs/epoxy nanocomposites

**Figure 1:**
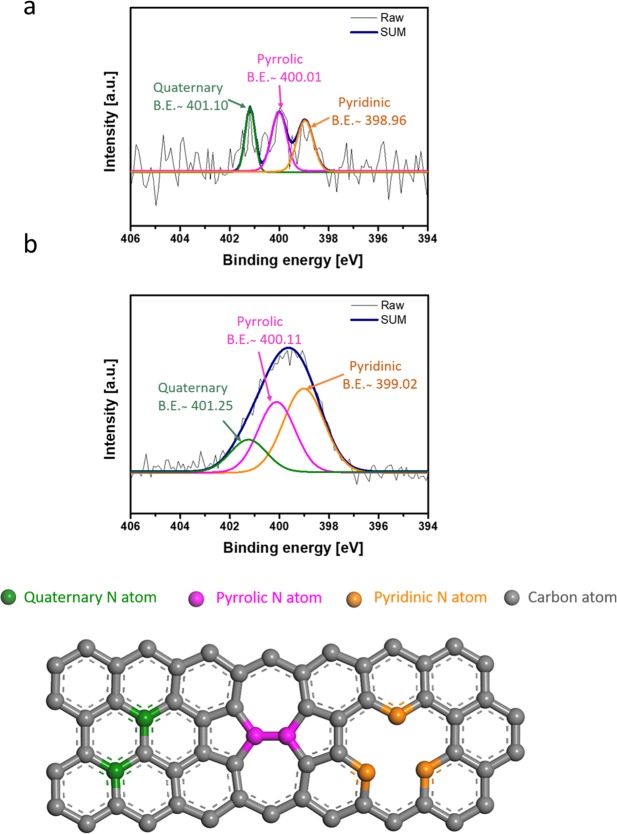
XPS N1s spectra survey from MWCNTs after nitrogen plasma treatments: **(a)** MSF-CNT, **(b)** ICP-CNT containing quaternary, pyrrolic, and pyridinic structures of nitrogen types.

The XPS spectra (Fig. 1(a,b)) of the nitrogen doped MWCNTs were recorded to identify the chemical composition of their surfaces. The N1s peak was found in the spectra of the MSF-CNT and ICP-CNT, which indicates that nitrogen was successfully doped onto the MWCNT surface. The nitrogen concentrations of the MSF-CNT and ICP-CNT were 1.5 and 4.0 atom (at) %, respectively. The ICP-CNT achieved nitrogen concentrations more than of three times greater than that of the low temperature, MSF-CNT. The plasma modification affected the contents and types of incorporated nitrogen in the doped MWCNT structures. The different N-types were distinguished with the aid of XPS. The spectra indicate to pyridinic nitrogen (398.3–399.8 eV), pyrrolic nitrogen (400.1–400.5 eV), quaternary nitrogen (401.0–401.4 eV) and to nitrogen oxide species and/or intercalated nitrogen molecules (404.0–405.6 eV)^22^. For the MSF-CNT, Fig. 1(a) shows spectra fitted with component peaks at 398.96 (10 at%), 400.01 (62 at%), and 401.10 eV (26 at%), respectively. The formation of different surface nitrogen groups was revealed by a detailed analysis of the N1s spectra.

**Figure 2:**
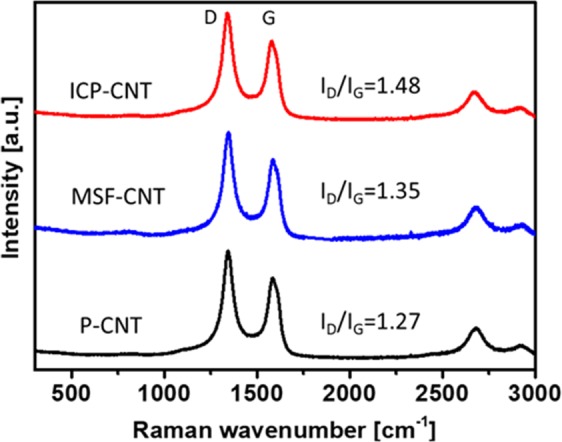
Raman spectra of pristine and modified MWCNTs.

Similarly, the N1s spectra of the ICP-CNT were fitted with component peaks at 399.02 (47 at%), 400.11 (36 at%), and 401.25 eV (16 at%). The MSF-CNT revealed a higher density of the pyrrolic type among nitrogen groups compared with that of the ICP-CNT. Moreover, the percent of pyrrolic types on the MSF-CNT was higher than those of the quaternary and pyridinic types. On the other hand, the percent of pyrrolic type nitrogen on the ICP-CNT was lower than that for the quaternary and pyridinic types. The survey spectra of the P-CNT only revealed the presence of carbon; notably, no traces of nitrogen were observed^22^. Nitrogen doping acts to modify the nanotube structure. Raman spectra was used to analyze the microstructure and defect level of the P-CNT, MSF-CNT, and ICP-CNT samples. The Raman spectra of the MWCNTs before and after the plasma treatment are exhibited in Fig. 2. At a high I_D_/I_G,_ many defects were present, which indicates that many groups appeared on the surface of the MWCNTs. The ratio of ICP-CNT was increased to 1.48 compared with the intensity ratio of the P-CNT (I_D_/I_G_ = 1.27), and MSF-CNT (I_D_/I_G_ = 1.35). This result represents that a few defects were produced on the surface of the ICP-CNT. Nitrogen-containing groups were successfully attached to the walls of the MWCNTs.

**Figure 3:**
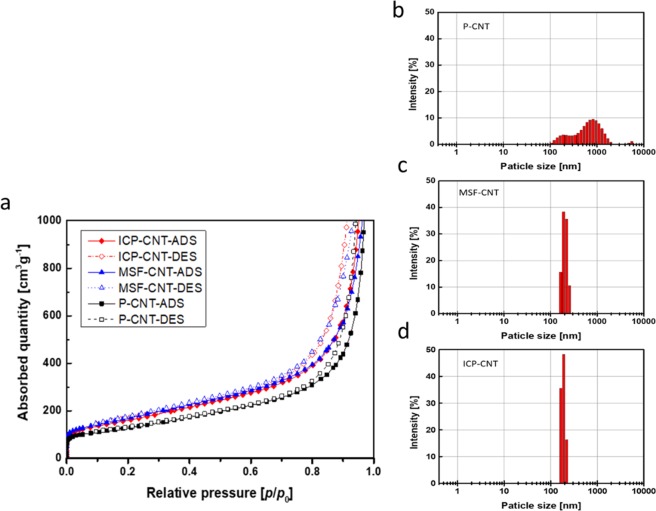
(**a**) BET-N_2_ adsorption/desorption isotherm of pristine and nitrogen doped MWCNTs. Zeta potential analysis of size distributions on MWCNT agglomerates in epoxy mixture containing filler of 2.0 wt%: **(b)** P-CNT, **(c)** MSF-CNT, and **(d)** ICP-CNT.

Nitrogen sorption analysis provided detailed information about the pore texture. The BET surface area and pore structure of the freeze-dried samples were analyzed by nitrogen physisorption, and the obtained isotherms are shown in Fig. 3(a). It can be seen that the isotherms of P-CNT, MSF-CNT, and ICP CNT are characteristic of the type IV shape^23^ with a distinct hysteresis loop in the low to high pressure regions (P/P_0_ = 0–1). The nitrogen doped CNTs (N-doped CNTs) prepared from different plasma treatments had a similar pore structure but with less pronounced hysteresis in isotherms. Overall, it can be concluded that all the samples had a typically mesoporous structure. The BET surface areas of P-CNT, MSF-CNT, and ICP-CNT were 498.38, 626.33, and 606.45 m^2^g^−1^. Notably, the specific surface area of the N-doped CNTs was higher than that of the P-CNT. The high surface area was likely due to the exposure of the inner surface of N-doped CNTs^24^. This is considered as a positive aspect because activated surface structure facilities heat conduction and therefore potentially enhances the heat dissipation when used in a light emitting diode package. The size and size distribution of the MWCNT aggregates, which were dispersed in the epoxy mixture after solvent-free processing, were measured by a particle size analyzer, as shown in Fig. 3(b–d). This Figure identifies any correlation between the MWCNTs dispersion and the thermo-mechanical properties of nitrogen doped nanocomposites. The P-CNT exhibit a trimodal distribution, with three peaks located at about 824.2 (77.7%), 204.3 (20.7%), and 5268 nm (1.6%). After nitrogen doping, both MSF-CNT and ICP-CNT show only a single distribution with average particle sizes of 203.6 and 185.8 nm, respectively. It is clear that the aggregate size indeed becomes smaller after nitrogen doping due to significantly weaker van der Waals interactions. Therefore, these nitrogen-doped MWCNTs are more easily dispersible. The ICP-CNT and MSF-CNT with nitrogen doping exhibited a higher dispersion level than the P-CNT. After nitrogen plasma modification, some nitrogen groups were attached to the surface of the MWCNTs, and improved the compatibility between the MWCNTs and epoxy resin, as well as the dispersion level of the MWCNTs. The mechanical properties of the nanocomposites are directly related to the state of dispersion of the reinforcements in the polymer matrix. When the reinforcements are poorly dispersed, the formations of nano or micro flaws result in local stress concentrations in the matrix. The brittleness of the composites is caused by these voids and defects, which result from MWCNT agglomerations. The thermal properties of a nanocomposite are dominated by atomic vibration or phonons and electrons. The agglomeration of MWCNTs block the phonon travels. Fig. S1 was used to analyze the dispersion of the modified MWCNTs (see Supplementary). Figures S1(b,c) indicate that excellent dispersion of the MWCNTs was achieved for all the epoxy nanocomposites containing nitrogen plasma treated MWCNTs.

**Figure 4:**
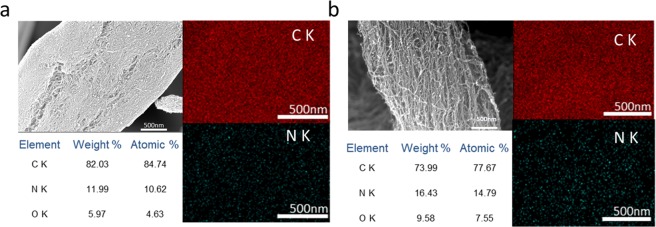
SEM images for EDAX analysis of the **(a)** MSF-CNT, **(b)** ICP-CNT.

**Figure 5:**
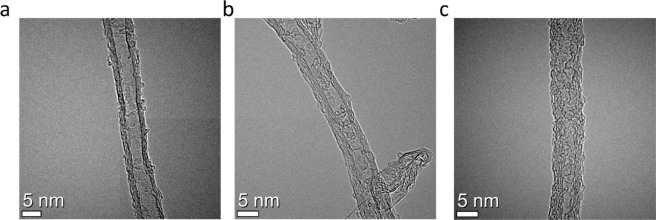
HR-TEM images for the **(a)** Pristine CNT, **(b)** MSF-CNT, and **(c)** ICP-CNT.

The SEM-EDAX analysis was performed to investigate distribution of different compositional elements for MSF-CNT and ICP-CNT. The amount of nitrogen observed in the ICP-CNT was approximately 14 at% (Fig. 4). This obtained value is higher than those of MSF-CNT. The HR-TEM micrographs in Fig. 5 demonstrate the presence of defects on the MWCNT surface. While pristine CNTs have a clean surface, ICP-CNT surface with higher the nitrogen doping content have roughness and many defects. It can be seen that the morphology of the CNT was successfully modified by nitrogen doping.

### Effect of the surface modification on thermo-mechanical properties

**Figure 6:**
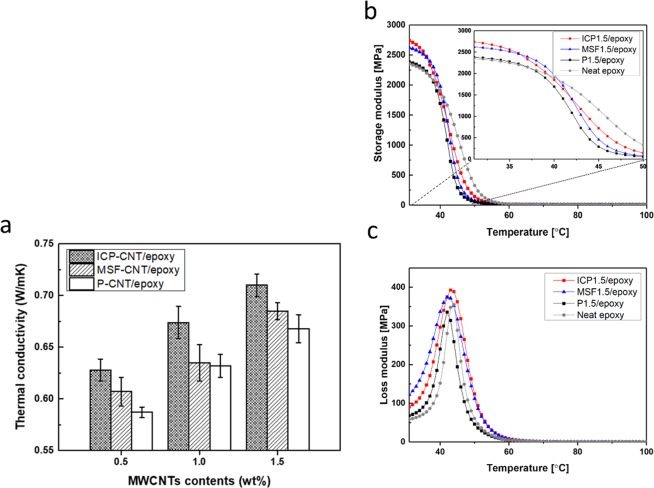
(**a**) Thermal conductivities of epoxy nanocomposites containing P-CNT, MSF-CNT, and ICP-CNT. Dynamic mechanical analysis of **(b)** storage modulus, *E*′ and **(c)** loss modulus, *E*″ of the neat epoxy composites and epoxy nanocomposite containing P-CNT, MSF-CNT, ICP-CNT.

The dynamic mechanical and thermal conductivity tests were performed to evaluate the effect of nitrogen doped MWCNTs on the thermal and mechanical properties of the composites. The thermal conductivities of P-CNT, MSF-CNT, and ICP-CNT reinforced epoxy nanocomposites are shown in Fig. 6(a) and Table 1 according to MWCNT content. The isotropic thermal conductivity of the nanocomposites containing P-CNT, MSF-CNT, and ICP-CNT steadily increased with an increasing content of MWCNTs. Increasing the content of MWCNTs reduces the matrix region between MWCNTs in a composite. This facilitates the interaction between MWCNTs with the enhancement of thermal conductivity. The increases in the thermal conductivity for the ICP-CNT/epoxy nanocomposites were consistently higher than those for P-CNT and MSF-CNT. It has been demonstrated that the presence of a large amount of pyridinic and quaternary (graphitic) nitrogen in the carbon network significantly enhances the activity performance of nitrogen doped CNTs in polymer composites^25^. The thermal conductivities of the nanocomposites containing ICP-CNT were increased by around 159–193% compared to the neat epoxy composites (0.242 W/mK) in the reported literature^26^. Similarly, the thermal conductivity of the nanocomposites containing MSF-CNT increased up to approximately 6.9% compared to the P-CNT/epoxy composites. The thermal properties of a nanocomposite are dominated by atomic vibration or phonons and electrons. The agglomeration of MWCNTs block the phonon travels. This phenomenon indicates that nitrogen modification improves the interfacial heat transport between the epoxy matrix and the MWCNTs. It also promotes better dispersion of MWCNTs in the matrix resulting to the enhancement of the thermal conductivity in MWCNTs/epoxy composites. DMA results are shown in Fig. 6(b,c). The storage modulus (*E*′) is a restitutive force that measures the energy storage capability of the material after elastic deformation. Figure 6(b) shows the storage modulus of the neat epoxy composites and nanocomposites containing the 1.5 wt% P-CNT, MSF-CNT, and ICP-CNT, which showed the most remarkable effect in the thermal properties. The ICP-CNT/epoxy nanocomposites had a higher modulus above and below the *T*_g_ (50 °C) compared to the P-CNT and MSF-CNT/epoxy nanocomposites. The storage modulus for P-CNT, MSF-CNT, and ICP-CNT reinforced epoxy nanocomposites were 2382, 2610, and 2739 MPa, respectively. The loss modulus (*E*″) is to the energy dissipated in the form of heat under deformation, i.e., the viscous response of the material. The loss modulus for P-CNT, MSF-CNT, and ICP-CNT reinforced epoxy nanocomposites were 335.8, 378.4, and 392.7 MPa, respectively. For the ICP-CNT/epoxy nanocomposite, the storage modulus was 14.9% and 4.9% higher than those of the P-CNT and MSF-CNT/epoxy nanocomposite at 30 °C. This significant increase in modulus for MSF-CNT and ICP-CNT/epoxy nanocomposites compared to P-CNT/epoxy nanocomposites was due to the strong interaction between the nitrogen group and the polymer, which led to restrictions on the mobility of the polymer chain. The applied stresses are expected to be easily transferred from the matrix to the MSF-CNT and ICP-CNT due to the high surface area of the nitrogen-doped fillers as shown the BET analysis. The addition of nitrogen doped CNTs reduced the chain mobility of the epoxy matrix, so that the thermal and mechanical properties were enhanced at high temperatures. The nitrogen group modification of CNTs is a significant factor determining the thermal and mechanical properties of the composite.

**Table 1:** Thermal properties of epoxy nanocomposites.

Sample (or molecular model)	Isotropic thermal conductivity, (W/mK)	Increase (%)	Longitudinal thermal conductivity (W/mK)	Transverse thermal conductivity (W/mK)
Neat epoxy^32^	0.242	—		
**P-CNT/epoxy**				
0.5 wt%	0.587 ± 0.5%	142		
1.0 wt%	0.632 ± 1.1%	161		
1.5 wt%	0.668 ± 1.3%	176		
1.5 wt% (MD)			5.752	0.280
**MSF-CNT/epoxy**				
0.5 wt%	0.607 ± 1.4%	150		
1.0 wt%	0.635 ± 1.7%	162		
1.5 wt%	0.685 ± 0.8%	183		
**ICP-CNT/epoxy**				
0.5 wt%	0.628 ± 1.0%	159		
1.0 wt%	0.674 ± 1.5%	178		
1.5 wt%	0.710 ± 1.0%	193		
1.5 wt% (MD^*1^)			6.223	0.270
1.5 wt% (MD^*2^)			8.176	0.310
1.5 wt% (MD^*3^)			7.292	0.328

^*^^1^: Pyrrolic type model, *^2^: Quaternary type model, *^3^: Pyridinic type model.

### Molecular dynamics simulations

The above results demonstrate the unique features of the nitrogen doped CNT reinforced epoxy composites: high dispersion stability and specific surface areas, modified nanotube structure, and enhanced thermal and mechanical properties. MD simulations were performed to estimate the effect of different nitrogen groups of the N-doped CNT surface on the thermal and mechanical properties. The three nitrogen doped CNTs were each modeled in the MD simulations. Additionally, the mechanical loading simulation details in the model were described in our previous study^22^. In Table 1, the longitudinal thermal conductivities were much higher than those of the transverse direction in all the nanocomposites models. The transverse directional thermal conductivities of Quaternary and Pyridinic were 10.7% and 17.1% higher than that of Pristine. This was due to a fact that while the longitudinal thermal conductivity of the nanocomposites depended on that of the CNTs, the thermal conduction in the transverse direction was significantly influenced by the interfacial thermal properties^27^. The longitudinal thermal conductivities of Quaternary and Pyridinic were 31.1 and 16.9% higher than that of Pyrrolic. Similarly, the transverse thermal conductivity of Quaternary and Pyridinic was 14.8 and 21.4% higher than that of Pyrrolic. This means that the nanocomposites remarkably exhibited an energy loss due to nitrogen doping with the pyrrolic type. This can be explained by considering the difference in defects and disrupted energy produced by the different nitrogen doping types. The results confirm that the nitrogen doped CNTs in nanocomposites may be another reason for dramatic enhancement of thermal properties according to nitrogen doping type. In the experimental analysis, the concentrations of pyrrolic type on the ICP-CNT was lower than those for the quaternary and pyridinic types. The isotropic thermal conductivity of nanocomposites containing ICP-CNT was higher than that of MSF-CNT. This explains its more favorable formation at higher nitrogen content. On the other hand, the pyrrolic nitrogen type, which includes pentagon defects, has been shown to lower energy in carbon nanostructures. Thus, an increased concentration of pyrrolic type nitrogen induces a larger number of defects on the carbon nanostructures. Therefore, controlling the type of nitrogen doping during the surface treatment of MWCNTs is critical to improving the thermal and mechanical properties of nanocomposites containing nitrogen doped MWCNTs.

## Discussion

This study investigated the thermo-mechanical properties of composite containing the modified MWCNTs with various types of nitrogen groups and an epoxy matrix. Two types of plasma treatments were performed, and both successfully modified the structures and morphologies of the MWCNTs. The obtained nitrogen doped CNTs containing mesoporous exhibited excellent actived surface structures. In addition, compared to the P-CNT, the ICP-CNT and MSF-CNT with nitrogen modification exhibited a higher dispersion level. The mechanisms were explained by effective energy transfer, and these conclusions were corroborated with the results of zeta potential size distribution studies, specific surface area, together with evidence from SEM analysis of the MWCNTs embedded in the matrix. The modified CNT surface structure may be due to the synergistic effect of the better dispersion, strong interfacial interaction, activity between the filler and matrix, and improved thermo-mechanical properties. In particular, the thermal conductivities of the nanocomposites containing ICP-CNT increased by around 3.4–6.1% compared to the MSF-CNT/composites. In the case of the tensile strengths, ICP-CNT/epoxy nanocomposites were 3.5–7.7% higher than those of the MSF-CNT/epoxy nanocomposites. The enhanced thermo-mechanical properties in the ICP/epoxy nanocomposites can be assigned to the higher content of quaternary and pyridinic types. The effects of different types of nitrogen doping were characterized from the experiments and MD models. Nanocomposite models containing quaternary and pyridinic nitrogen doping types showed higher thermal and mechanical properties than that of an MD model containing pyrrolic types. These trends matched the experimental values well. Controlling the types of nitrogen on MWCNTs is critical to improving the thermo-mechanical properties of nitrogen doped MWCNT composites.

## Method

### Materials and preparation of physical modified MWCNTs/epoxy nanocomposites

**Figure 7:**
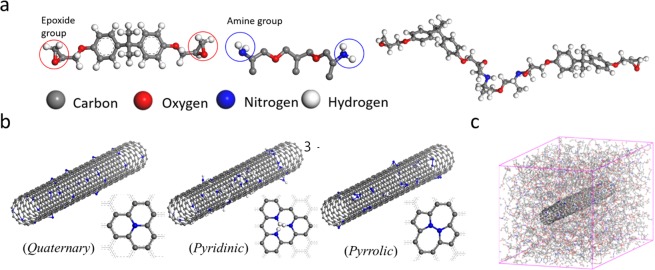
(**a**) Chemical structures of the epoxy precursors and cured networks. Molecular structures of MD simulations. **(b)** nitrogen group doped CNTs and **(c)** relaxed nanocomposite after ensemble.

Multi-walled carbon nanotubes (MWCNTs) with lengths of 10–50 μm were produced by a Catalytic Chemical Vapor Deposition (CCVD) process with a purity of more than 95% carbon (JEIO Co., Ltd., Korea). The epoxy used in the study was EPON 826, which has a low viscosity, diglycidyl ether of bisphenol A (DGEBA) (Momentive Specialty Chemicals Inc., USA) and the 50 wt% curing agent was the aliphatic diamine, Jeffamine D-230 (Huntsman Co., Houston, TX). Figure 7(a) illustrates the molecular structures of the epoxy precursor and curing agent.

**Figure 8:**
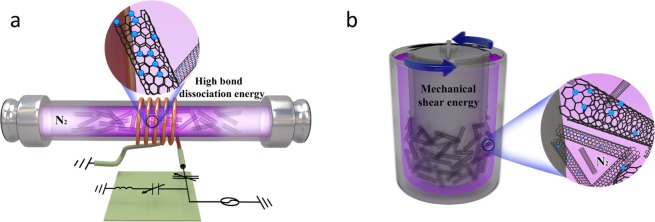
Schematic diagrams for the nitrogen plasma treatment process: **(a)** Inductively coupled plasma and **(b)** Mechanical shear forced plasma.

Plasma treatments of the surface of MWCNTs can be performed at an atmospheric pressure in order to lower costs and an increase of the speed of the process. To compare the doping effects in the two different environments, the MWCNTs were modified using two different plasma process chambers. Figure 8 schematically describes the formation of nitrogen plasma treatment process. First, an effective method of nitrogen doping using environmentally friendly Inductively coupled plasma (ICP) and the optimization for manufacturing epoxy-based nanocomposites were mentioned from our previous study^22^. Second plasma treatment employed mechanical shear forces and a plasma apparatus (Nanocular NC, HOSOKAWA Micron Ltd., Japan). This process technology creates direct solid bonding between particles using mechanical shear force and additional physical plasma energy without any binders^28^. The press head in the chamber rotates at high speed to provide intensive interactions with the coating powders. The present study used untreated MWCNTs (P-CNT) and MWCNTs modified through Inductively coupled plasma (method 1, ICP-CNT) and mechanical shear force (method 2, MSF-CNT) prepared using the noted nitrogen plasma treatments. Epoxy nanocomposites containing 0.5, 1.0, and 1.5 wt% of P-CNT, ICP-CNT, or MSF-CNT were manufactured.

### Characterizations

The influence of physical modifications and structural defects on the nitrogen doping concentration of MWCNTs was examined using X-ray photoelectron spectroscopy (XPS, K-Alpha, Thermo Fisher Scientific, Inc., USA) and Raman spectroscopy (Renishaw Invia Raman microscope, Renishaw, England). The 514 nm excitation line of an argon laser was used, while the laser power was measured directly on the specimen. Three spectra were recorded per sample. The specific surface area of MWCNTs was determined with a physisorption analyzer (Belsorp-Max, MicrotracBel Co., Japan) using the Brunauer-Emmett-Teller (BET) methods at −196 °C. The dispersion stability of the MWCNTs in the matrix was evaluated by measuring the epoxy mixture after dilution in ethanol, using a particle size analyzer (Zetasizer Nano ZS, Malvern Instruments Ltd., Australia) based on the dynamic light scattering (DLS) technique. The thermomechanical properties of the composites were measured using a DMA Q800 dynamic mechanical analyzer (TA Instruments, USA) from the single cantilever mode at 1 Hz frequency from 30 to 250 °C with a heating rate of 1 °C min^−1^. The specimen dimensions were 17.2 mm × 8.6 mm × 2.7 mm. The thermal conductivity analyzer (TPS 2500 S, Hot Disk AB, Gothenburg, Sweden) was used to measure the isotropic (three-dimensional randomly oriented) thermal conductivities of the nanocomposites according to the ISO 22007–2^29^. The tensile properties of the MWCNTs/epoxy composites were measured using universal testing machine (Unitech-M, R&B, Korea) with an extensometer (Model 3542, Epsilon Tech. Corp., USA) by 1 mm min^−1^ cross-head speed according to ASTM D 638 (see Supplementary Table S1)^30^. Twenty specimens were tested for each set of conditions and their standard deviations were calculated. The dispersion level of the MWCNTs and the interfacial quality between the MWCNTs and the matrix were observed using a scanning electron microscope (FE-SEM, Nova NanoSEM 450, FEI Corp., OR, USA). The samples were observed from Scanning Electron Microscope and Energy Dispersive X-ray microanalysis (SEM-EDAX, Verios 460) to analysis the concentration and distribution of different compositional elements. A High Resolution Transmission Electron Microscope (HR-TEM) FEI model TECNAI G2 F30 instrument operated at an accelerating voltage of 300 kV was used for analyzing the structure and morphology of the MWCNTs.

### An equilibrium molecular dynamics

An equilibrium molecular dynamics (EMD) simulation was used to obtain the thermal conductivities of the nanocomposites containing CNTs. The thermal conductivity vector (*k*_*i*_) using the Green-Kubo relation is expressed as^31^

1 $${k}_{i}=\frac{1}{V{k}_{b}{T}^{2}}{\int }_{0}^{\infty }\langle {J}_{i}(t)\cdot {J}_{i}(0)\rangle dt,$$

where *V* is the volume of the MD model, *k*_*b*_ is the Boltzmann constant (=1.38 × 10^−23^ J/K), and *T* is inner temperature of the MD model. *J*_*i*_(*t*) is the heat current vector at time *t*. $\langle \rangle$ indicates an autocorrelation function. The heat current vector is expressed as

2 $${J}_{i}(t)=\mathop{\sum }\limits_{j}^{N}\{{e}_{j}{v}_{j}+\frac{1}{2}\sum _{j < l}{f}_{jl}{r}_{jl}({v}_{j}+{v}_{l})\},$$

where *N* is the total number of atoms, *e*_*j*_ and *v*_*j*_ are the total energy and velocity of the *j* ^th^ atom, respectively. *f*_*jl*_ and *r*_*jl*_ are the interatomic force and position vector between the *j* ^th^ and *l* ^th^ atoms.

The molecular model of the CNT is a single-walled structure with a diameter of 0.79 nm. Four percent of surface atoms on the CNT model were replaced by defects. The defects were randomly located on the CNT surface. The other CNT models consisted of three different nitrogen doping groups, quaternary, pyridinic, and pyrrolic. The nitrogen doping groups were formed on the defects. The ratio of nitrogen to carbon atoms for the three nitrogen doped CNT models was 0.04. Polymer chains for the matrix were made by crosslinking two unit epoxy (Epon 826) chain and hardener (Jeffamine D230) molecules. The composite models were constructed by dispersing the polymer chains around the CNT model with a density of 1.13 g cm^−3^ (Fig. 3(b,c)). The composite model containing the CNTs with defects is denoted by ‘Pristine’. The models containing the three different nitrogen doped CNTs are denoted by ‘Quaternary’, ‘Pyridinic’, and ‘Pyrrolic’, according to the nitrogen doping type. The energy minimization of the four composite models using a conjugate algorithm was performed to stabilize their potential energies. To relax the energy minimized models, the NPT (isobaric-isothermal state, N: constant number of atoms, P: constant pressure, T: constant temperature) ensemble was carried out at room temperature and under atmospheric pressure during 1 ns. The relaxed models of the four different composites were used to calculate their thermal conductivities from the EMD simulations. The NVE (microcanonical state, N: constant number of atoms, V: constant volume, E: constant potential energy) ensemble was simulated for 2 ns. The heat current vector, volume, and temperature were calculated at each time step of 1 fs during the NVE process. The thermal conductivity was obtained by substituting the calculated values in Eqs. (1) and (2). In addition, the Dreiding force field has been widely used for MD simulations of biological, inorganic, and organic materials. It consist of the forcefield constants for analyzing thermal conduction of pristine CNTs, nitrogen doped CNTs, and various polymer chains in MD simulations. It also has a strong point that can analyze a hydrogen bonding energy as well as common non-bonding energies such as van der Waals and coulombic. This is important for accurately analyzing the non-bonding interactions between CNTs and polymer chains. Hence, the LAMMPS (Large-scale Atomic/Molecular Massively Parallel Simulator)^32^ and the Dreiding force field^33^ were used in every MD simulation.

## Supplementary information

Supporting Information.

Supporting Information 1.
