# Supplementary material for: Enhancement of thermo-mechanical stability for nanocomposites containing plasma treated carbon nanotubes with an experimental study and molecular dynamics simulations
Source: Sci Rep. 2020 Jan 15;10:405. doi: 10.1038/s41598-019-56976-w (PMC6962151; doi:10.1038/s41598-019-56976-w)
Supplement: Supplementary file 1 — Supporting Information. [file 41598_2019_56976_MOESM1_ESM.docx]

**Supplementary Information**

**Enhancement of thermo-mechanical stability for nanocomposites containing plasma treated carbon nanotubes with an experimental study and molecular dynamics simulations**

Hana Jung, Hoi Kil Choi, Yuna Oh, Hyunkee Hong, Jaesang Yu*

Composite Materials Application Research Center, Institute of Advanced Composite Materials, Korea Institute of Science and Technology (KIST),

* Corresponding author: Jaesang Yu

E-mail: jamesyu@kist.re.kr


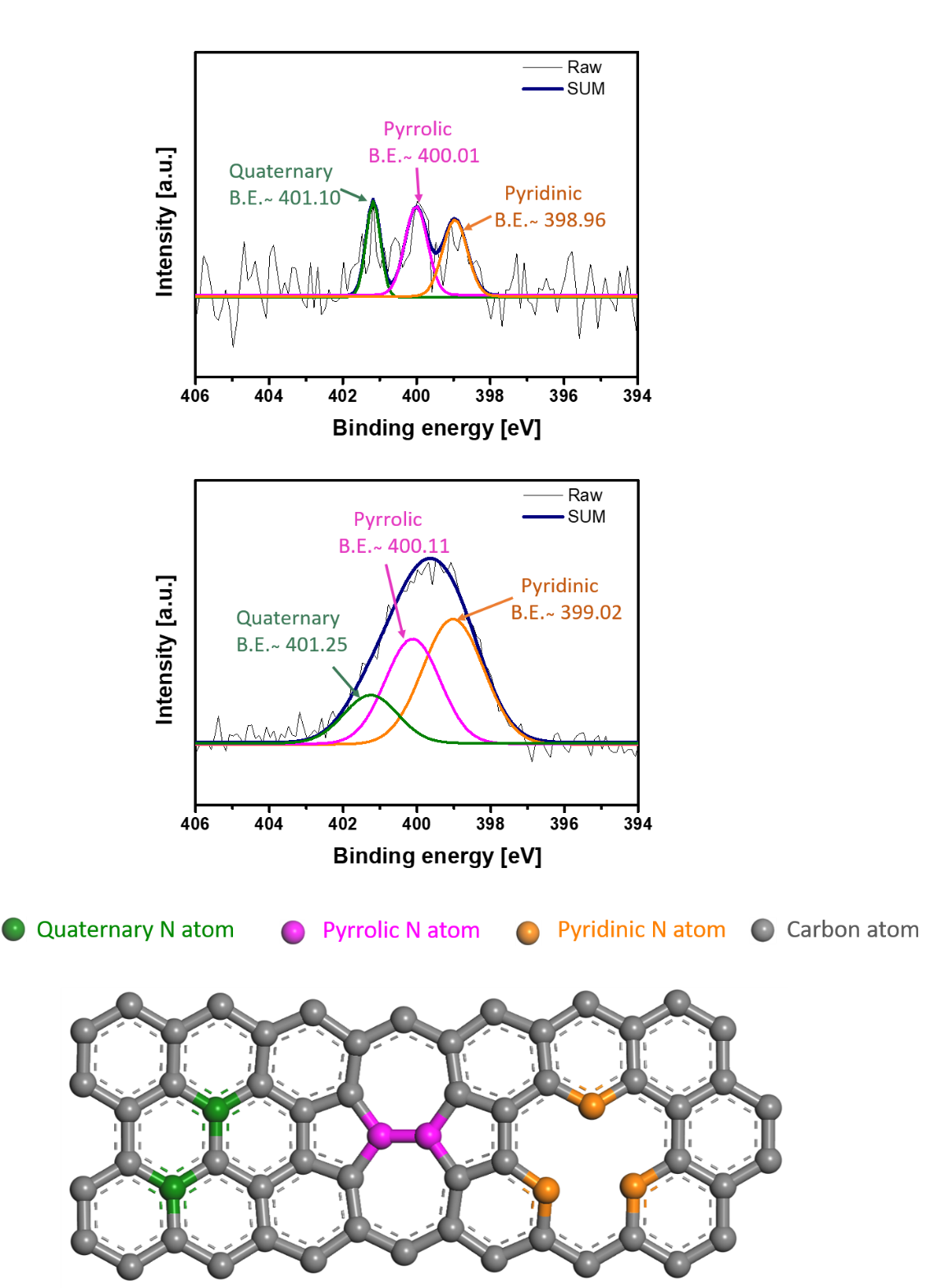
**Appendix A. Morphologies of the pristine and nitrogen doped MWCNTs**

(a)

(b)

**Figure S1.** XPS N1s spectra survey from MWCNTs after nitrogen plasma treatments: (a) MSF-CNT, (b) ICP-CNT containing quaternary, pyrrolic, and pyridinic structures of nitrogen types.

**
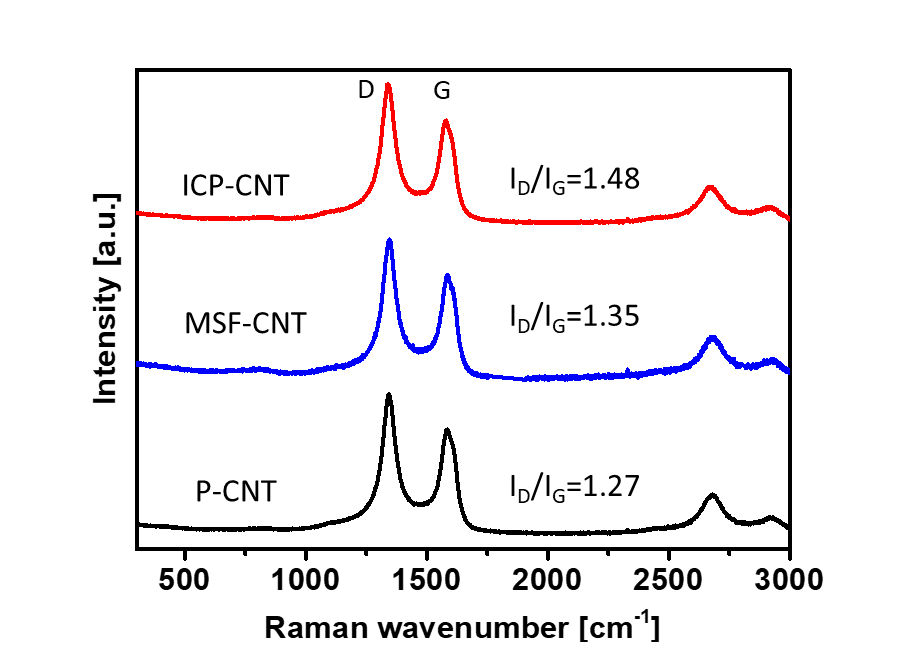
**

**Figure S2.** Raman spectra of pristine and functionalized MWCNTs.

**Appendix B. Mechanical properties of epoxy nanocomposites containing P-CNT, MSF-CNT, and ICP-CNT.**

**Table S1** Tensile properties of epoxy nanocomposites

| Sample  (or molecular model) | Elastic modulus,  *E* (GPa) | Increase  (%) | Tensile strength,  *TS* (MPa) | Increase  (%) |
| --- | --- | --- | --- | --- |
| Neat epoxy | 2.57±0.05 | - | 37.53±0.05 | - |
| P-CNT/epoxy |  |  |  |  |
| 0.3 wt% | 2.78±0.08 | 8.2 | 41.2±1.5 | 9.8 |
| 0.6 wt% | 2.83±0.19 | 10.1 | 35.3±1.4 | -5.9 |
| 1.0 wt% | 2.79±0.09 | 8.6 | 33.1±1.1 | -11.8 |
| 1.0 wt% (MD) [22] | 3.83 | 49.0 | - | - |
| MSF-CNT /epoxy |  |  |  |  |
| 0.3 wt% | 2.90±0.09 | 12.8 | 41.3±1.9 | 10.0 |
| 0.6 wt% | 2.92±0.09 | 13.6 | 36.8±7.2 | -1.9 |
| 1.0 wt% | 2.94±0.08 | 14.4 | 34.9±2.2 | -7.0 |
| ICP-CNT/epoxy [22] |  |  |  |  |
| 0.3 wt% | 2.97±0.09 | 15.6 | 42.9±1.2 | 14.3 |
| 0.6 wt% | 2.94±0.02 | 14.4 | 38.1±4.8 | 1.5 |
| 1.0 wt% | 2.97±0.05 | 15.6 | 37.6±2.1 | 0.2 |
| 1.0 wt% (MD^*1^) | 3.78 | 47.1 | - | - |
| 1.0 wt% (MD^*2^) | 4.12 | 60.3 | - | - |
| 1.0 wt% (MD^*3^) | 3.99 | 55.3 | - | - |

*1: pyrrolic type model, *2: quaternary type model, *3: pyridinic type model

Mechanical properties were measured to evaluate the effect of the nitrogen functionalized MWCNTs on the mechanical properties of the composites. The mechanical behavior on the nanocomposites containing P-and ICP-CNT were referred to our previous study [22]. Table B.1 presents the tensile properties as a function of MWCNT contents for the different nitrogen plasma modifications, together with P-CNT. The elastic moduli of the P-CNT, MSF*-*CNT, and ICP-CNT reinforced epoxy composites were increased compared to that of the neat epoxy. All the tensile properties of the MSF-CNT/epoxy composites were superior to those of the P-CNT/epoxy composites. The elastic moduli of the MSF-CNT/epoxy nanocomposites increased up to approximately 14.4 % compared to that of the neat epoxy. These increases were higher than those of the P-CNT/epoxy nanocomposites. As previously discussed, the uniform dispersion of the ICP-CNT and MSF-CNT can prevent the formation of flaws, which is disturbed to stress transfer from reinforcements to the matrix. The nitrogen plasma modification was expected to improve the dispersion of the MWCNTs as well as their interfacial interaction. The nitrogen doping groups can cause the polarity effects such as reduced work-function and produced band gap energy [22]. The mechanical properties of the nitrogen doped CNT reinforced nanocomposites were improved compared to those of the P-CNT/epoxy nanocomposites. That means the MSF-CNT and ICP-CNT should have the stronger interfacial bonding with the epoxy resin in comparison to P-CNT. Evidently, this effect accounts for the good interfacial interaction of the functionalized MWCNTs, as shown in Fig. B.1 (e-f). The MWCNTs are well embedded in the matrix. The excellent interfacial interaction between the MWCNT and the surrounding polymer matrix plays an important role for effective stress transfer. In contrast, the P-CNT/epoxy composite in Fig. B.1 (d) exhibits poor interaction between the reinforcements and the matrix. P-CNTs in the epoxy matrix were poorly dispersed at higher weight fractions of MWCNTs. The behavior of nano-sized reinforcements in the matrix are generally complex issues related to load transfer, stress concentration, and defect distribution. In the case of the ICP-CNT/epoxy composites, the tensile strengths were 3.5~7.7 % higher than those of the MSF-CNT/epoxy composites. This means that the higher nitrogen concentrations of the MWCNTs effectively enhanced the mechanical properties of the composites. According to this study, it can be concluded that the introduction of nitrogen functionalized MWCNTs improved the interfacial bonding with the epoxy matrix resulting to the profound improvement in the mechanical properties of composites.

In Table B.1, the elastic modulus of the 1.0 wt% P-CNT/epoxy composite calculated by MD simulation is 37.1% higher than the experimentally measured value. Also, the elastic moduli of the 1.0 wt% MSF- and ICP-CNT/epoxy composites measured from experiments were lower than those obtained from the MD models for nanocomposites containing 1.0 wt% functionalized MWCNTs. These results are due to differences in the dispersion of the MWCNTs in the specimens and the MD models. The MD models have a large total interphase area between the epoxy matrix and MWCNTs, since the dispersion of the MWCNTs was uniform in the MD models. The calculated elastic moduli of the MD models for nanocomposites containing 1.0wt% MWCNTs functionalized by quaternary and pyridinic nitrogen types (quaternary and pyridinic MD models) were 7.8 and 4.5% higher than that of the MD model for the nanocomposite containing 1.0wt% P-CNT (a pristine MD model). On the other hand, the calculated elastic moduli of the MD models for nanocomposites containing 1.0wt% MWCNTs functionalized by pyrrolic nitrogen types (pyrrolic MD models) was 1.0% lower than that of the pristine MD model. These results mean that the nanocomposites that contain MWCNTs functionalized by quaternary and pyridinic nitrogen types were more rigid against deformation than those containing MWCNTs functionalized by pyrrolic type.

(a)
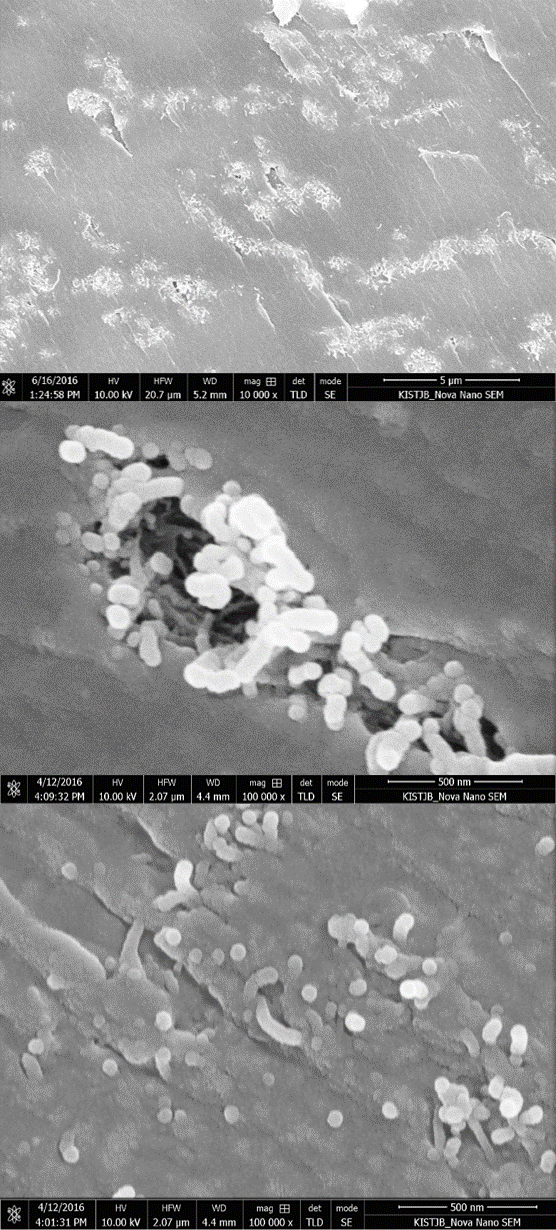

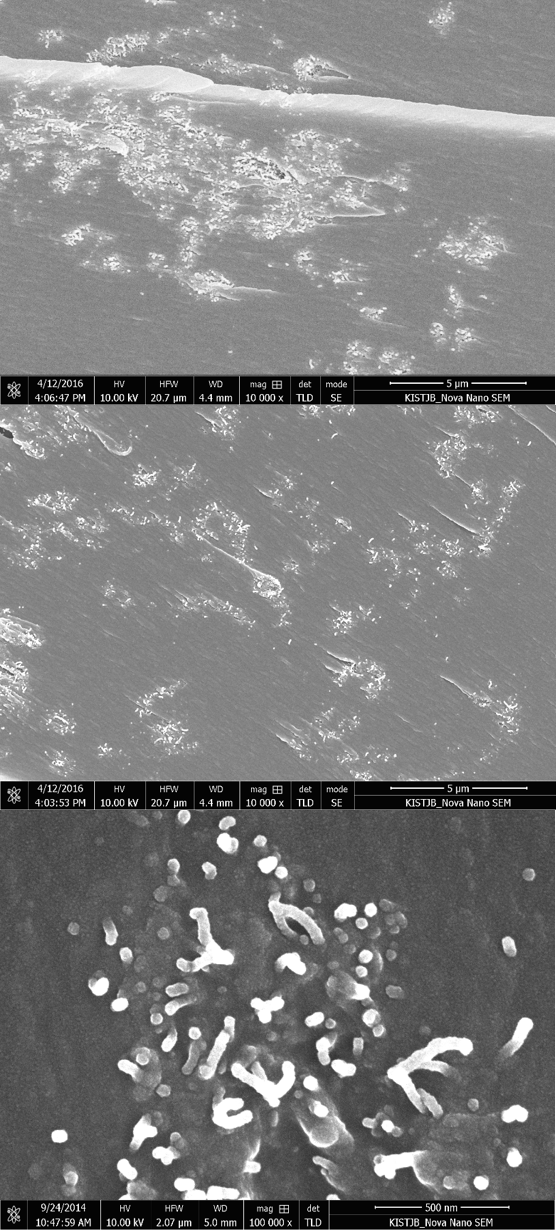
 (b)

(c) (d)

(e) (f)

**Figure S3.** FE-SEM images of the fracture surfaces of the epoxy composites containing of the 1.0 wt% (a) P-CNT, (b) MSF-CNT, and (c) ICP-CNT at high magnification ×10,000 and (d) P-CNT, (e) MSF-CNT, and (f) ICP-CNT [22] at high magnification × 100,000.
